# Association of systemic and ocular risk factors with neurosensory retinal detachment in diabetic macular edema: a case–control study

**DOI:** 10.1186/1471-2415-14-47

**Published:** 2014-04-09

**Authors:** Aditi Gupta, Rajiv Raman, Vaitheeswaran Kulothungan, Tarun Sharma

**Affiliations:** 1 Shri Bhagwan Mahavir Department of Vitreoretinal Services, Sankara Nethralaya, 18, College Road, Chennai 600 006, Tamil Nadu, India; 2 Department of Preventive Medicine and Biostatistics, Sankara Nethralaya, 18, College Road, Chennai, 600 006 Tamil Nadu, India

**Keywords:** Diabetic macular edema, Neurosensory retinal detachment, Risk factors, Blood Pressure

## Abstract

**Background:**

Diabetic macular edema (DME) with neurosensory retinal detachment (NSD) remains an important cause of visual loss in patients with diabetes. The aim of the study was to elucidate the association of systemic and ocular risk factors with NSD in DME.

**Methods:**

In a retrospective case–control study, we reviewed clinical records of all the subjects with DME seen between January 2010 and December 2010. Cases and controls were selected based on optical coherence tomography and stereoscopic biomicroscopy review. NSD was defined as subfoveal fluid accumulation under detached retina with or without overlying foveal thickening. The association between the presence of NSD, blood pressure, lipid status and various other biochemical parameters was evaluated.

**Results:**

Group I (cases) included 37 eyes of 33 patients having DME with NSD and Group II (controls) included 30 eyes of 21 patients having DME without NSD. Patients ranged in age (mean ± SD) from 50 to 62 years (56.6 +/-6.78) for cases and from 51 to 65 years (58.4+/-7.84) for controls. The duration of diabetes ranged from 4 to 15 year (mean 9.45+/-6.08) among cases and 4 to 14 years (9.7+/-5.12) among controls. Significant risk factors for NSD were high values of systolic and diastolic blood pressure (p = 0.039 and 0.043 respectively).

**Conclusion:**

High systolic and diastolic blood pressures are independent and significant risk factors for NSD in DME.

## Background

Diabetic macular edema (DME) remains a major cause of visual loss in patients with diabetes [1]. Optical coherence tomography (OCT) has specifically been used for characterizing the morphological features of DME, and five OCT patterns of DME have been described: diffuse retinal thickening (DRT), cystoid macular edema (CME), neurosensory retinal detachment (NSD) without posterior hyaloidal traction, posterior hyaloidal traction (PHT) without tractional retinal detachment (TRD) and PHT with TRD [2-5]. NSD under the fovea has been reported in 3–31% of patients with DME [2,4-10].

The prognosis of DME is decided by many factors, such as the presence of NSD, inner segment/outer segment (IS/OS) conjunction and integrity of the external limiting membrane (ELM) line [11-13]. IS/OS integrity and intact ELM are important indicators in the evaluation of foveal photoreceptor layer integrity, and correlate strongly with best-corrected visual acuity (BCVA) after medical or surgical treatment of DME [11,12].

The presence of NSD is found to adversely affect the prognosis of DME. NSD can significantly limit effective laser treatment of the macula [14]. The presence of NSD in DME associated with subretinal exudation has been reported to be associated with poor visual prognosis after vitrectomy [2]. Likewise, in macular edema secondary to branch retinal vein occlusion, the presence of subfoveal NSD was shown to retard the absorption of macular edema and recovery of vision after grid laser photocoagulation [14]. The high percentage of NSD in CRVO [9] may have played a role in the poor response of macular edema to grid laser photocoagulation in the multicenter trial on CRVO by the Central Retinal Vein Occlusion Study Group [15]. Hence, the need to better understand the pathogenesis of NSD has been stressed [15]. Although previous studies have extensively reported the systemic and ocular risk factors for the presence of DME [16-20], the risk factors associated with NSD in DME have been rarely studied in detail [21]. A recent study indicated the presence of high glycosylated hemoglobin (HbA1c) as a risk factor for NSD, suggesting the role of systemic factors in the causation of NSD in DME [21]. The aim of this study was to elucidate the association of various systemic and ocular risk factors with NSD in Indian subjects with DME.

## Methods

**Figure 1:**
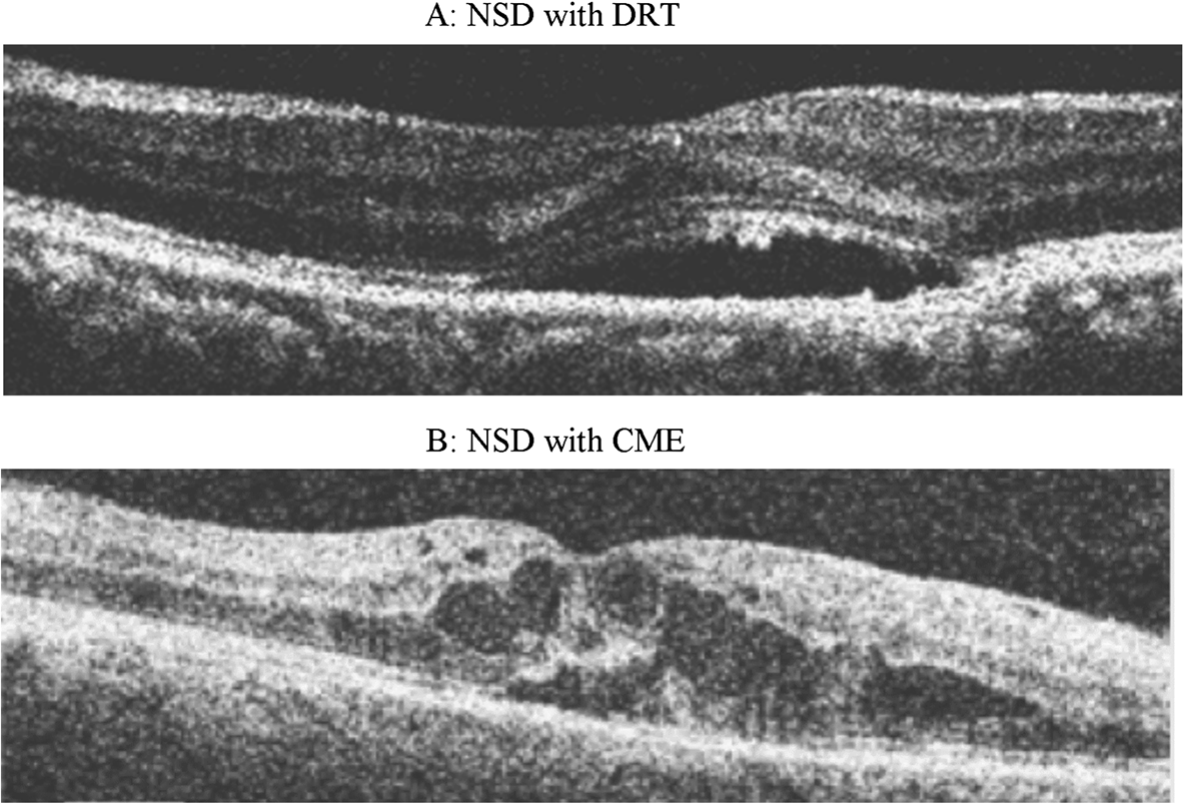
Spectral domain OCT shows diffuse retinal thickening (A) and hyporeflective cystic spaces in the inner retina suggestive of cystoid macular edema (B) with associated subfoveal neurosensory detachment.

**Figure 2:**
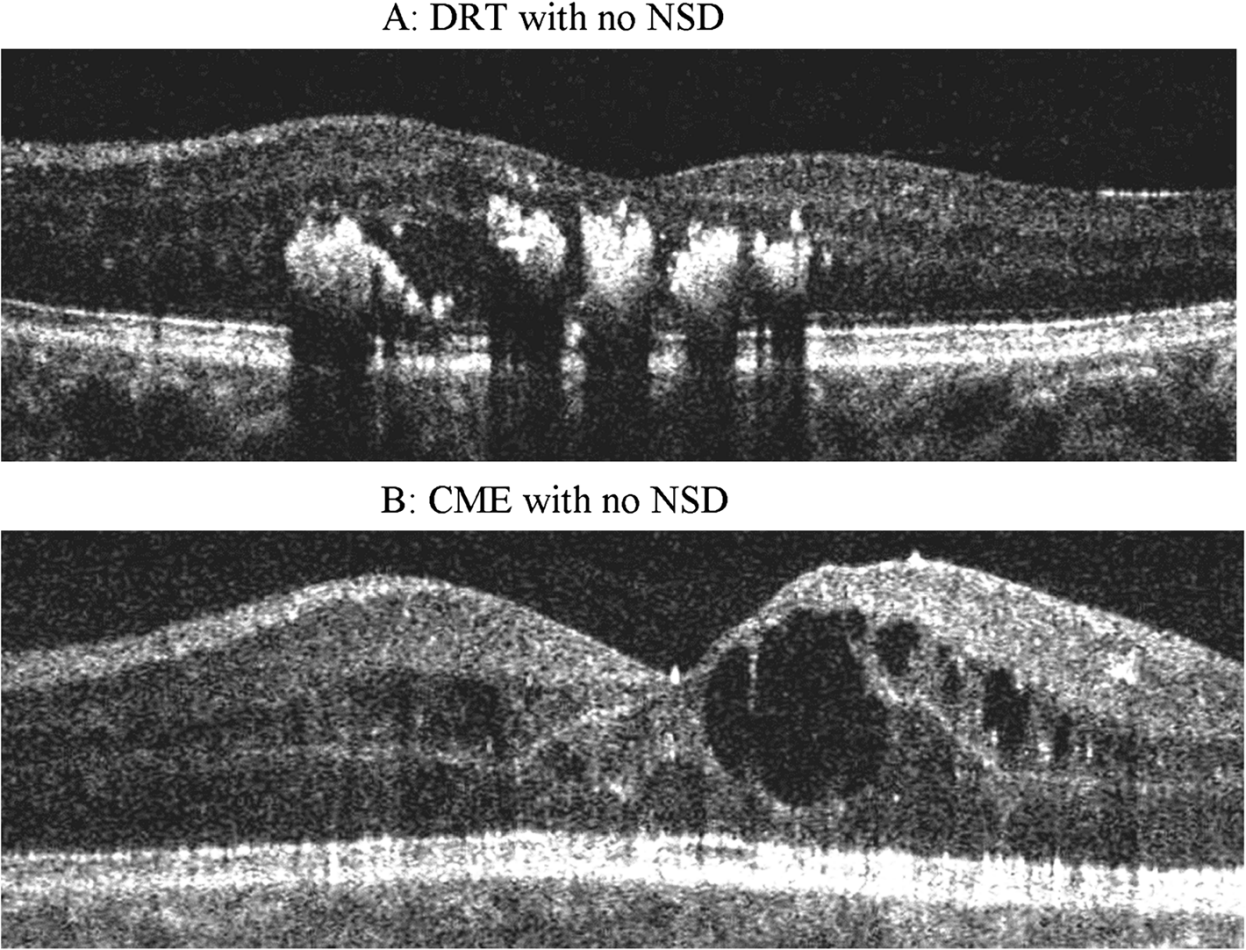
Spectral domain OCT shows diffuse retinal thickening (A) and hyporeflective cystic spaces in the inner retina suggestive of cystoid macular edema (B) without any neurosensory detachment.

This study was a retrospective chart review of patients with diagnosed DME seen between Jan 2010 to Dec 2010. Cases and controls were selected based on SD-OCT review. Group I (cases) included 37 eyes of 33 patients who were diagnosed as DME with NSD on OCT and Group II (controls) included 30 eyes of 21 patients who had DME without NSD. The macular edema was diagnosed by biomicroscopy according to the criteria reported by ETDRS. NSD type DME was defined as subfoveal fluid accumulation with distinct outer border of detached retina with or without overlying foveal thickening (Figure 1). All cases had NSD associated with DRT or CME (Figure 1). All controls had DRT or CME without any NSD (Figure 2). Patients with posterior hyaloidal traction (PHT) without TRD and PHT with TRD as documented on OCT, and media opacities such as corneal opacity, dense cataract, vitreous or preretinal hemorrhage, uveitis were excluded from the study. This study was approved by the Institutional Review Board, Vision Research Foundation and adhered to the Declaration of Helsinki. The medical records of all the patients including cases and controls were reviewed, with documentation of patients’ age, gender, duration and type of diabetes mellitus, history of any associated systemic disease like hypertension, hyperlipidemia, nephropathy, ischemic heart disease status post coronary artery bypass surgery, details of systemic medications, history of ocular surgeries including intravitreal injections and laser treatment in the past. The details of patients’ ocular examination findings including BCVA on Snellen chart (later converted to logMAR for statistical analyses), lens status, stage of diabetic retinopathy, OCT findings, and systemic biochemical parameters were also noted. A prototype SD-OCT system (Topcon 3D1000, Tokyo, Japan) was used with an axial resolution of 6u and acquisition rate of approximately 18,000 scans per second. All OCT images were acquired through a dilated pupil. During the OCT examination, macula was scanned on six radial sections including the horizontal, vertical and oblique planes through the centre of the fovea. The retinal thickness was measured as distance of vitreoretinal interface and inner edge of retinal pigment epithelium (RPE) at the maximum point of edema. Central subfield retinal thickness was also noted on OCT.

The blood pressure was recorded, in the sitting position, in the right arm to the nearest 2 mm Hg using the mercury sphygmomanometer (Diamond Deluxe BP apparatus, Pune, India). Two readings were taken with a five minutes interval and their mean indication was taken as the blood pressure. In most of the subjects, the blood pressure measurement was taken on the day of complete ophthalmic examination which included OCT. In the remaining subjects, blood pressure was measured on the day of OCT review which was 1 or 2 days later. The biochemical parameters noted were fasting and post-prandial blood sugar levels, hemoglobin, glycosylated hemoglobin, blood pressure and renal function tests (serum urea and serum creatinine). All biochemical parameters were done at same laboratory using standard techniques. The grading of diabetic retinopathy was done based on modified klein classification as mild, moderate and severe nonproliferative diabetic retinopathy and proliferative diabetic retinopathy [22]. The modification was proposed as a standardized alternative to the more detailed Early Treatment Diabetic Retinopathy Study (ETDRS) system. It involves grading seven stereoscopic standard fields as a whole, and assigning a level of severity for the eye according to the greatest degree of retinopathy using a modified Airlie House Classification scheme [22].

### Statistical analysis

A computerized database was created for all the records. Statistical analyses were performed using SPSS Windows version 14.0 (SPSS Science, Chicago, IL, USA). All the data were expressed as mean ± S.D or as percentage. The normality of distribution was checked for all factors by Kolmogorov–Smirnov analysis. The data in the study followed normal distribution, hence we used the parametric tests to determine significance. Chi-square test was used to compare proportions among neurosensory detachment status with the independent categorical variables and the Student’s *t*-test was used to compare proportions among neurosensory detachment status with the independent measured (continuous) variables in Univariate analyses. P value less than 0.05 was considered significant. Multivariate analyses could not be done because of small sample size in both the groups.

## Results

A total of 37 eyes of 33 patients who had NSD with DME (Group 1, cases) and 30 eyes of 21 patients who had DME without NSD (Group 2, controls) were included in our study. All patients included were of type 2 diabetes mellitus. The patients ranged in age from 50 to 62 years among the cases (mean 56.6 +/-6.7) and 51 to 65 among the controls (mean 58.4+/7.84). The duration of diabetes ranged from 4 to 15 years (mean 9.45+/-6.08) among cases and 4 to 14 years (9.7+/-5.12) among controls. Group 1 included 28 males (84.8%) and 5 females (15.2%) and Group 2 included 14 males (67.7%) and 7 females (33.3%).

Table 1 depicts the comparison of systemic factors associated with cases and controls. There were no significant differences in the two groups in terms of the mean age (p = 0.375), duration of diabetes (p = 0.876), fasting blood sugar and post prandial blood sugar levels (p = 0.959 and 0.436 respectively), glycosylated hemoglobin (p = 0.859), hemoglobin (p = 0.118) and presence of hypertension (p = 0.634). However, the mean systolic and diastolic blood pressures were significantly higher (p = 0.039 and 0.043 respectively) in the NSD group than the control group.

**Table 1:** Comparison of systemic features amongst cases and controls

	**Cases (33 patients)**	**Controls (21 patients)**	**P value**
**Age (years)**	56.6+/-6,78	58.4 +/-7.84	0.375
**Gender n (%)**			
Males	28 (84.8%)	14 (66.7%)	
Females	5 (15.2%)	7 (33.3%)	
**Duration of diabetes (years)**	9.45 +/- 6.08	9.7 +/-5.12	0.876
**Systemic associations n (%)**			
Hypertension	24 (72.7%)	14 (66.7%)	0.634
Hyperlipidemia	3 (9.1%)	1 (4.8%)	1.000
Nephropathy	1 (3%)	0	1.000
S/p CABG	1 (3%)	0	1.000
**Systemic medications n (%)**			
Antidiabetic insulin	3 (9.1%)	3 (14.3%)	0.667
Group 1 (biguanides)	7 (21.2%)	9 (42.9%)	0.089
Group 2 (glitazones)	5 (15.2%)	1 (4.8%)	0.386
Group 3 (sulfonylureas)	22 (66.7%)	14 (66.7%)	1.000
Antihypertensive	18 (54.6%)	10 (47.6%)	0.619
**Mean value of systemic parameters**			
HbA 1c	6.73 +/- 1.42	6.71 +/- 1.39	0.859
SBP (mm Hg)	147.84 +/- 11.33	141 +/- 11.88	**0.039**
DBP (mm Hg)	85.24 +/-5.98	81.47 +/- 7.29	**0.043**
FBS (mg%)	144.05 +/- 81.59	142.94 +/- 70.42	0.959
PPBS (mg%)	229.17 +/- 112.52	208.15 +/- 60.97	0.436
Urea	37.82 +/- 17.0	34.52 +/- 19.0	0.509
Creatinine	1.41 +/- 0.74	1.07 +/- 0.331	0.053
Hemoglobin (g%)	10.71 +/- 2.36	11.77 +/- 2.44	0.118

S/P CABG: Status post coronary artery bypass graft, HbA1c: Glycosylated hemoglobin, SBP: Systolic blood pressure, DBP: Diastolic blood pressure, FBS: fdasting blood sugar, PPBS: Post prandial blood sugar.

P value <0.05 considered significant (boldface).

Table 2 compares the ocular factors among eyes with NSD and control eyes. The mean log MAR BCVA was 0.822 +/-0.421 in NSD group and 0.61+/-0.47 in the control group (p 0.056). The mean central macular thickness as determined by OCT was higher in cases than in the controls (485.7 +/-189.81 versus 332.2+/-134.14 microns, p = 0.0004). Cystoid macular edema was more commonly seen than diffuse edema in NSD group, although the difference was not significant (p = 0.085). 35.1% of eyes with NSD had associated proliferative diabetic retinopathy compared to 23.3% in the control group (p = 0.820). There were no significant differences in the two groups in terms of pseudophakic status (p = 0.370). As expected, ocular surgery including anti- VEGF injections were performed more frequently in Group 1 (p = 0.008).

**Table 2:** Comparison of ocular findings amongst cases and controls

	**Cases (37 eyes)**	**Controls (30 eyes)**	**P value**
**Mean BCVA (logMAR)**	0.822 +/- 0.421	0.61+/- 0.470	0.056
**Mean CMT (microns)**	485.7 +/- 189.81	332.2 +/- 134.14	**0.0004**
**Type of edema n (%)**			
CME	25 (67.6%)	14 (46.7%)	0.085
Diffuse	12 (32.4%)	16 (53.3%)	
**Stage of DR n (%)**			
Mild NPDR	0	6 (20%)	0.820
Moderate NPDR	15 (40.5%)	15 (50%)	
Severe NPDR	9 (24.3%)	2 (6.7%)	
PDR	13 (35.1%)	7 (23.3%)	
**Phakic status n (%)**			
Phakic	33 (89.2%)	29 (96.7%)	0.370
Aphakic	0	0	
Pseudophakic	4 (10.8%)	1 (3.3%)	
**Ocular surgery** (includes anti VEGF injections) n (%)	19 (51.4%)	6 (20%)	**0.008**
**S/P PRP n (%)**	12 (32.4%)	6 (20%)	0.254

Bcva: Best corrected visual acuity, CMT: Central macular thickness, CME: Cystoid macular edema, DR: Diabetic retinopathy, NPDR: Non proliferative DR, VEGF: Vascular endothelial growth factor, S/P PRP: status post pan retinal therapy.

P value <0.05 considered significant (boldface).

## Discussion

DME remains the leading cause of visual loss among patients with diabetes mellitus. Among the various patterns of DME, NSD under the fovea has been reported in 3–31% of patients. The pathogenesis of NSD is linked not only to the limitations of the draining vascular system, but also to impairment in the function of the RPE. Kang et al. reported that in diabetic eyes, the incidence of CME and NSD increasd with the existence of retinal vascular hyperpermeability and the pathology of these two phenomena might share a common pathogenesis in this regard [4].

Various systemic factors have been associated with increased incidence of DME like severity of diabetic retinopathy, poor glycemic control and duration of diabetes. Hypertension, proteinuria, dyslipidemia, uncontrolled renal parameters, and PRP for PDR (causing acute choroidal ischemia), have also been associated with increased risk of DME. Although all these factors are known to correlate with increased incidence of DME, very few studies have correlated the presence of uncontrolled systemic disease and biochemical parameters with increased incidence of NSD in DME. Poor control of systemic factors could be related to increased leakage from the capillaries with loss of vascular integrity as well to an impaired function of RPE. One study demonstrated the presence of high HbA1c levels in the patients with diabetic CME and NSD, compared to those with diabetic CME and no associated NSD [21]. In our study, we did not find a significant association between HbA1c and presence of NSD. Instead, only high mean systolic and diastolic blood pressures were found to be independent and significant risk factors for NSD in DME.

Increased blood pressure has been implicated, through the effects of increased blood flow, to cause damage to the retinal capillary endothelial cells in eyes of diabetic patients [23]. Elevated blood pressure also alters the retinal arteriolar hemodynamics, causing a reduction in the compliance (i.e., an increase of vascular rigidity) of the arteriolar circulation with increasing risk of DME [20]. Hypertension is a well recognized cause of NSD preferentially affecting the macular region, although NSD is more commonly accompanied with malignant hypertension [24,25]. The occurrence of NSD in DME can be secondary to excessive leakage in retina or to a poorly functioning RPE. Raised blood pressure can lead to increased retinal leakage as well as ischemic damage to RPE. Another possibility is that diabetes may have caused subclinical choroidal vascular damage in diabetic subjects, rendering the circulatory system more susceptible to further ischemic insult by raised blood pressure.

Choroidal vascular damage causes ischemic damage to the RPE and leads to breakdown of the blood-retinal barrier with transudation of fluid into subretinal space. Hayreh observed that the presence of NSD was correlated to the degree of choroidal circulation disruption. Fluid overload has also been implicated as a cause of NSD [26].

Anemia is another known risk factor for DME. Low hemoglobin levels can occur in diabetic patients secondary to renal disease or can occur independently. However, the renal disease as measured by serum urea and creatinine was not found to be associated with NSD in this study. Futhermore, anemia was not found as an independent risk factor for formation of NSD. Low hemoglobin has been described as an independent baseline risk factor in the EDTRS for the development of DME and severe visual loss [27]. Other studies have corroborated this finding [18] and have also found improvement in the DME status following correction of anemia [28-30]. Correction of anemia (and also supplementation of erythropoietin) was noted to decrease the effects of retinopathy with structural improvement, possibly through improved oxygenation of the macula [28]. Singh et al. noted spontaneous closure of microaneurysms in diabetic retinopathy with treatment of co-existing anemia [30]. Friedman et al. reported that increased hematocrit may improve visual acuity due to resolution of macular edema in diabetic retinopathy [29]. Over the past few years, growing evidence supports the hypothesis that hypoxia contributes to progression of tissue injury in diabetic individuals [31]. In our study, although the patients with NSD had lower hemoglobin, none of them had significantly low hemoglobin levels which could be clinically called as anemia. This could be the reason why we were unable to find any significant effect of anemia on NSD.

## Conclusion

In conclusion, we found high systolic and diastolic blood pressures to be independent and significant risk factors for NSD in DME. The present study suggests that the treating ophthalmologists should get a complete systemic workup done for the presence of co-morbidities especially high blood pressure in subjects with NSD in DME. Achieving adequate metabolic control of associated conditions should be aimed in such subjects. Prospective studies are warranted to see whether decreasing the blood pressure of the patient will help in the resolution of NSD in DME.

## Competing interests

The authors declare that they have no competing interests.

## Authors’ contributions

AG carried out the data collection, data analysis, and drafted the manuscript. RR conceived the study, and participated in its design and coordination and helped to draft the manuscript. VK helped in data collection, data analysis and performed the statistical analysis. TS helped to conceive the study, supervised the entire study and helped to draft the manuscript. All authors read and approved the final manuscript.

## Pre-publication history

The pre-publication history for this paper can be accessed here:

http://www.biomedcentral.com/1471-2415/14/47/prepub
